# Identified the novel resistant biomarkers for taxane-based therapy for triple-negative breast cancer

**DOI:** 10.7150/ijms.59177

**Published:** 2021-04-26

**Authors:** Ching-Wen Chou, Yu-Min Huang, Yu-Jia Chang, Chien-Yu Huang, Chin-Sheng Hung

**Affiliations:** 1 Graduate Institute of Clinical Medicine, College of Medicine, Taipei Medical University, Taipei, Taiwan, ROC.; 2 Department of Obstetrics and Gynecology, National Taiwan University Hospital, Taipei, Taiwan.; 3 Department of Surgery, School of Medicine, College of Medicine, Taipei Medical University, Taipei, Taiwan, ROC.; 4 Section of General Surgery, Department of Surgery, Taipei Medical University Hospital, Taipei, Taiwan, ROC.; 5 Cell Physiology and Molecular Image Research Center, Wan Fang Hospital, Taipei Medical University, Taipei, Taiwan.; 6 Department of Pathology, Wan Fang Hospital, Taipei Medical University, Taipei, Taiwan.; 7 Division of General Surgery, Department of Surgery, Shuang Ho Hospital, Taipei Medical University, New Taipei City, Taiwan, ROC.; 8 Division of Colonrectal Surgery, Department of Surgery, Shuang Ho Hospital, Taipei Medical University.

**Keywords:** TNBC, GEO, taxane, bioinformative, basal-like-TNBC.

## Abstract

Developing treatment strategies for triple-negative breast cancer (TNBC) has become an important clinical challenge. Currently, taxane-based chemotherapy is one of the standard treatments for TNBC. However, determining the key factor of taxane-resistance is urgently in need for clinical treatment for breast cancer. We used GEO data to generate paclitaxel resistance in two basal-like TNBC cell lines (SUM149 and MDA-MB-468). Seventy-one common upregulated differentially expressed genes (DEGs) and 11 downregulated DEGs were found to be related to paclitaxel resistance. By constructing protein-protein interactions, 28 hub proteins with a degree cutoff criterion of ≥1 were found. Nine hub genes (*COL4A6*, *COL4A5*, *IL6*, *PDGFA*, *LPAR1*, *FYB*, *IL20*, *IL18R1* and *INHBA*) are involved in important signaling pathways**.** We found that upregulated *PDGFA* and downregulated *COL4A6* were significantly associated with an insensitive response to neoadjuvant paclitaxel-based therapy. A Kaplan-Meier plot was created to check the prognostic values of 11 hub DEGs in terms of recurrence-free survival. High expressions of *PDGFA* and *LAMB3* were correlated with poor recurrence-free survival, while low levels of *FYB*, *IL18R1*, and *RASGRP1* indicated poorer relapse-free survival. Our results suggest that *PDGFA*, *COL4A6*, *LPAR1*, *FYB*, *COL4A5*, and *RASGRP1* might be candidate target genes for taxane-based therapy in basal-like TNBC.

## Introduction

Breast cancer is the most common malignancy in women worldwide. It is a heterogeneous disease with different clinical behaviors, morphological and genetic characters 1. Therefore, breast cancers could be classified by immunohistochemistry (IHC) using such as progesterone receptor (PR), estrogen receptor (ER), and human epidermal growth factor receptor (HER)-2 as molecular markers for clinical use 2. The subtypes categorized by IHC are luminal A, luminal B, basal-like and Her2/neu. The recent developed gene expression classifier, PAM50, defined by mRNA analysis of 50 genes, is important for therapeutic reference. PAM50 include five subtypes, Luminal A, Luminal B, Basal, Her2-enriched and normal-like 3. Triple-negative breast cancer (TNBC) means the absence of ER and PR expressions and a lack of amplification of the *HER-2* gene 4, 5. TNBC occurs in around 12%~25% of cases of breast cancer and is aggressive in younger, African-American women and familial breast cancer susceptibility gene (BRCA)-mutated breast cancer 6-8. Patients with TNBC have a poorer prognosis than the patients of other subtypes. In general, single or sequential single-agent cytotoxic chemotherapy is the common option for TNBC, but therapeutic failure is seen in more than 50% of TNBC patients (but the overall survival rate shows no difference) 9. In March 2019, USA FDA approved the combination use of both immunotherapy and chemotherapy agents, Atezolizumab and Nab-paclitaxel, for the beneficial in OS outcome. In April 2020, USA FDA approved the use of Trodelvy (chemical name: sacituzumab govitecan), an immunotherapy medicine, to treat metastatic TNBC patients since the progression free survival and overall survival were better based on the results of clinical phase III trial. The use of pembrolizumab along with chemotherapy treatment for the patients with locally recurrent unresectable or metastatic TNBC. negative breast cancer (TNBC) whose tumors express PD-L1 (CPS ≥10).

There are six distinct TNBC subtypes defined by analysis of messenger (m)RNA expressions, including basal-like (BL-1, BL-2, and IM), claudin-low (M and MSL), and molecular apocrine cancers (LAR) 10. Basal-like cancers are the most common variant in TNBC, which account for approximately 70-80% of TNBC patients, exhibit progenitor-like or luminal/myoepithelial features, whereas claudin-low cancers show a significant mesenchymal phenotype 11. Later, IM and MSL subtypes were found contributed from infiltrating lymphocytes or any tumor-associated stromal cells, respectively. Therefore, the classification was refined from six into four tumor-specific subtypes as BL1, BL2, M, and LAR 12.

TNBC is the most aggressive subtype and has been one of the most difficult issues of breast cancer in terms of overall poor outcomes after treatment 13, 14. Taxane-containing cytotoxic regimens (e.g., paclitaxel and docetaxel) are the main treatments for TNBC 15. Taxanes are antineoplastic agents that acts by disrupting the microtubular network in cells that is essential for mitotic and interphase cellular functions. Taxanes binds to free tubulin and promote the assembly of tubulin into stable microtubules while simultaneously inhibiting their disassembly. This leads to the production of microtubule bundles without normal function and to the stabilization of microtubules, which results in the inhibition of mitosis in cells. 16. The most effective regimens for TNBC currently reach only 40-45% pathologic complete response (pCR) rates, which are achieved by taxane/anthracycline sequential treatment or combination of taxane with the platinum drugs 17. Previous reports showed little or no improvement in pCR rates of other chemotherapy drugs such as capecitabine, gemcitabine, ixabepilone, or vinorelbine 17-19. There is no valid biomarker or effective treatment target to predict taxane resistance in TNBC 9, 20. Even in patients of the early stages, high systemic relapse rates and mortality after metastasis remain key obstacles 21, 22. Various mechanisms associated with resistance to taxane-based chemotherapy have been proposed, including overexpression of the ATP-binding cassette drug efflux transporters (ABCB1) or of the multidrug resistance protein (MRP1) and of the breast cancer resistance protein (BCRP, ABCG2) 23, 24. Furthermore, previous studies revealed that CYP3A/2C, enzymes of the cytochrome P450 subfamily, play a vital role to metabolize taxane anticancer agents which can affect the intrinsic taxane susceptibility of these tumors 25. Although there are some genomic features shared by all kinds of TNBC, individual subtypes may contribute to different clinical outcomes 26. It remains an urgent need for novel therapies for taxane-resistant TNBC.

In this study, we used the bioinformatic dataset to find out the possible candidate targets for taxane-resistance in TNBC. The expression dataset from paclitaxel-resistant cells were used to identify differentially expressed genes (DEGs) and constructed a protein-protein interaction network. The functional clustering analysis and clinical responses were integrated to identify significant key DEGs and important pathways involved. Finally, recurrence free survival analysis was performed to verify the patients' outcome. We found that *PDGFA*, *COL4A6*, *LPAR1*, *FYB*, *COL4A5*, and *RASGRP1* might be candidate target genes for taxane-based therapy for basal-like TNBC. The information may provide a more in-depth understanding for taxane resistance related genetic heterogeneity in basal-like TNBC subtypes and for determining key genes related to chemotherapeutic failure.

## Materials and methods

### mRNA expression datasets

The GSE90564 and GSE25065 datasets were downloaded from the Gene Expression Omnibus (GEO; http://www.ncbi.nlm.nih.gov/geo/) database at the National Center of Biotechnology Information (NCBI) and analyzed with the GEO2R tool (https://www.ncbi.nlm.nih.gov/geo/geo2r/). Data derived from GSE90564 using the GEO2R analysis consisted of 62973 probe sets. In addition, the GSE90564 mRNA dataset contained five different cell lines: BT20, SUM149, MDA-MB-231, MDA-MB-436, and MDA-MB-468. MDA-MB-468 is a BL1 cell line, while SUM149 belongs to the BL2 subtype. In addition, MDA-MB-231 and MDA-MB-436 are respectively classified as the MSL and LAR subtypes. BT20 was not classified. Because the cell lines mentioned above carry unique characteristics individually, and around 70-80 % of TNBCs being the basal-like subtype 11, we focused on analyzing MDA-MB-468 and SUM149, both of which are basal-like subtypes in this study. Each group contained three to four parental and resistant cell lines. The expression profile of was detected using an Agilent-028004 SurePrint G3 Human GE 8x60K Microarray platform.

The GSE25065 dataset (total 310 patients) contained 31 TNBC patients who were treated with 12 cycles of paclitaxel weekly with each administration being before or after 4 cycles of anthracycline-based therapy. All TNBC patients in this dataset belong to clinical stage II-III. The clinical data showed that five TNBC patients were sensitive to paclitaxel-based treatment, while 26 patients were insensitive. Sensitive response is defined as pathological complete response or minimal residual cancer burden. The microarray data were obtained by using the Affymetrix Human Genome U133A Array.

### Identification of DEGs

From the mRNA dataset, DEGs were identified through the GEO2R application using Benjamini & Hochberg statistics to calculate the *p* value. The screening threshold was set to *p*<0.05 and absolute log2 (fold change) of >1. In the analysis of GSE90564, we focused on identifying paclitaxel-resistant biomarkers of MDA-MB-468 and SUM149. First, we identified DEGs by comparing resistant cell lines with their parental cell lines individually. Then, upregulated DEGs were intersected between MDA-MB-468 and SUM149 to deliver common genes with upregulated expressions, and likewise for common downregulated DEGs.

### Construction of a protein-protein interaction (PPI) network

STRING vers. 10.0 (http://www.string-db.org/) was used to construct the PPI networks. This is a web-based biological database for predicting known and unknown protein interaction relationships. A combined score of >0.7 (high confidence) was selected as the cutoff criterion. Then, PPI pairs were input into Cytoscape software version 3.4.0 (http://www.cytoscape.org) to construct the PPI network. Common DEGs (up- and downregulated ones) were used for the PPI network analysis. Then the CytoNCA app for cytoscape was used to calculate the degree (the number of lines connecting the proteins), and we identified nodes with a degree cutoff criterion of ≥1, viewed as hub proteins, which were simultaneously differentially expressed between parental cell lines and paclitaxel-resistant cell lines.

### Functional cluster analysis

To identify highly connected proteins (hub nodes) with important biological functions, we utilized the Database for Annotation, Visualization and Integrated Discovery (DAVID) (https://david.ncifcrf.gov) to provide genomic functional annotations and predict possible pathways. In this study, a gene ontology enrichment analysis was performed utilizing DAVID. An enrichment score of >1 was set as the criterion to identify significant functional clusters. Interconnected proteins (hub nodes) with important biological functions were viewed as candidate genes potentially associated with paclitaxel resistance. The screening threshold was set to p-value <0.05. We also utilized Benjamini-Hochberg correction to calculate adjusted p-value which was corrected by false discovery rate approach.

### Clinical validation of DEGs

We validated expression levels of important hub genes by comparing five paclitaxel- sensitive patients and 26 paclitaxel-insensitive patients in the GSE25065 dataset. A receiver operating characteristic (ROC) curve for each possibly important DEG was calculated to present the sensitivity, specificity, and accuracy of predicting responses to paclitaxel or docetaxel. In order to assess recurrence-free survival of the 11 important candidate DEGs which were predicted to be associated with taxane resistance, we utilized an online survival analytical tool to rapidly assess the effects of gene expressions on TNBC prognosis 27. This online tool was established to analyze survival information of 1809 breast cancer patients downloaded from the GEO, which utilized the Affymetrix HGU133A and HGU133+2 microarrays. In this study, we analyzed 186 TNBC/basal-like patients who had undergone chemotherapeutic treatment. The log-rank test was applied to identify the significance of differences between subgroup-regulated high and low gene expression levels. These two groups were divided by the median expression level. In addition, the hazard ratio was calculated to reveal associations of gene expressions with recurrence-free survival.

## Results

### Target DEGs in paclitaxel-resistant basal-like TNBC cells

**Figure 1:**
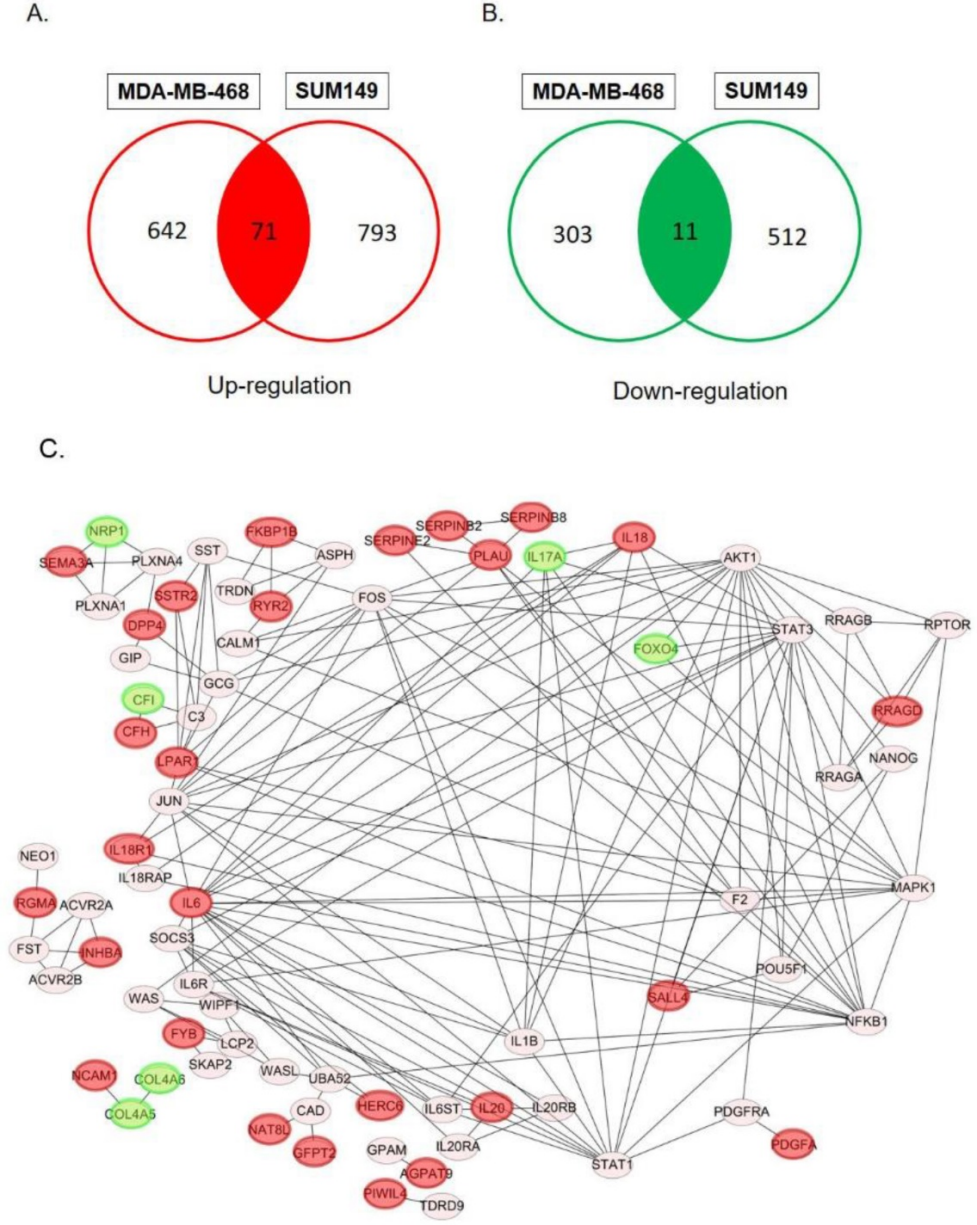
Venn diagram of overlapping differentially expressed genes (DEGs) in paclitaxel-resistant and basal-like triple negative breast cancer cells: (A) 71 common upregulated DEGs and (B) 11 downregulated DEGs between the MDA-MB-468 and SUM149 cell lines. These DEGs met the criteria of the absolute log2 (multiple of change) >1 and *p*<0.05 we set. (C) Construction of protein-protein interaction (PPI) networks and identification of hub proteins. We utilized 82 differentially expressed genes (DEGs) to predict protein interactions using STRING software and obtained 79 PPI pairs. All of the significantly (*p*<0.05) correlated protein networks were mapped, and the red and green nodes denote upregulated and downregulated DEGs, respectively. In total, 28 hub genes were identified as proteins with a degree cutoff of ≥1 and were simultaneously differentially expressed between parental cell lines and paclitaxel-resistant cell lines.

In order to determine specific genes contributed to paclitaxel-resistant outcomes in basal-like TNBC cells, we used the GSE90564 mRNA dataset which contained data on five different TNBC cell lines, including parental and paclitaxel-resistant gene expression patterns. We compared expression levels between parental and paclitaxel-resistant SUM149 and MDA-MB-468 cells and found 82 candidate DEGs, including 71 upregulated and 11 downregulated DEGs (Fig. 1A, B, Supplementary Tables 1, 2), which may be involved in paclitaxel resistance in basal-like TNBC cell lines.

### PPI networks

**Figure 2:**
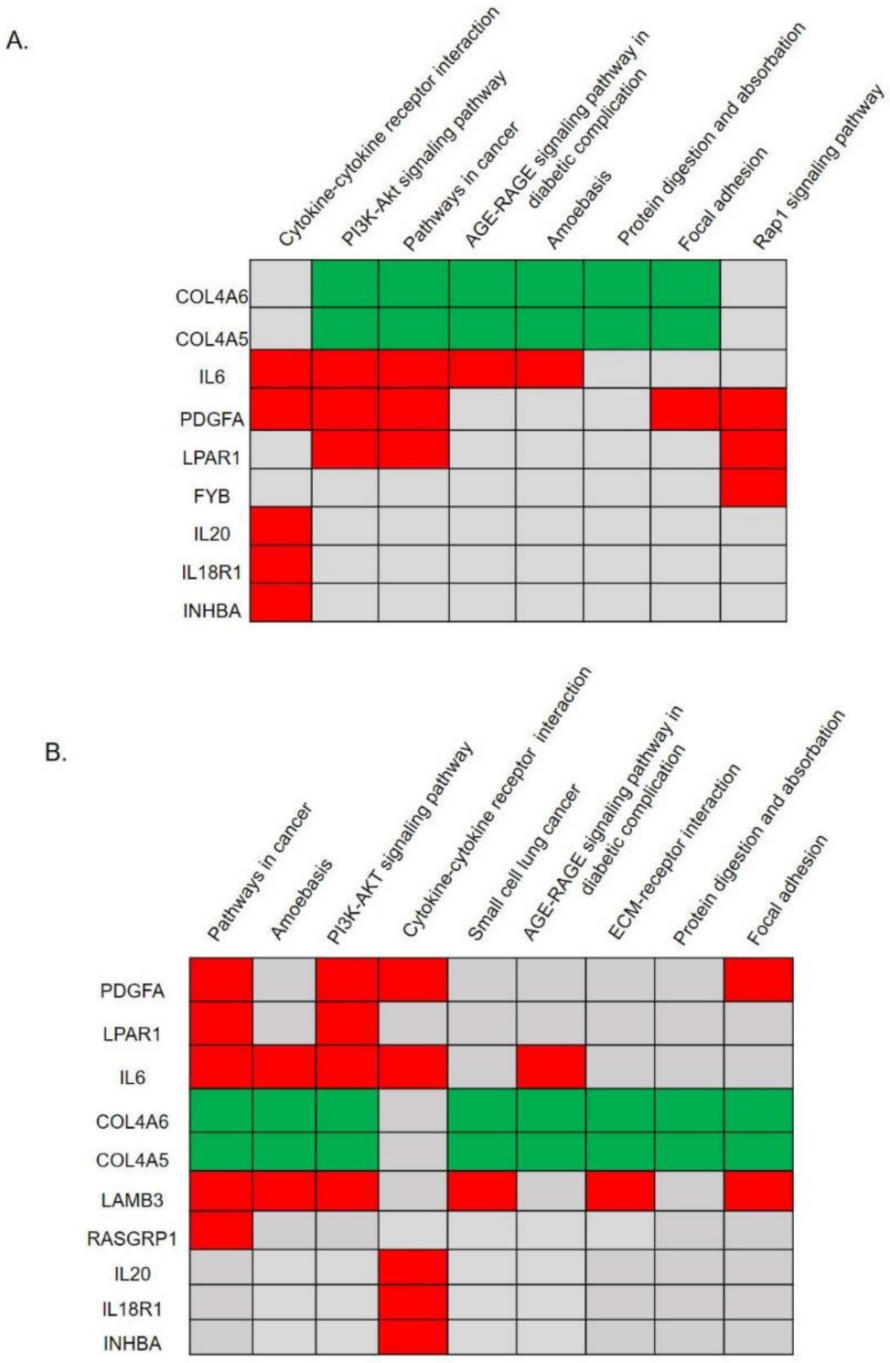
Significant KEGG pathway predictions by DAVID. (A) Predicted signaling pathways of 28 candidate genes with a protein-protein interaction network analysis. The top nine genes of *COL4A6*, *COL4A5*, *IL6*, *PDGFA*, *LPAR1*, *FYB*, *IL20*, *IL18R1*, and *INHBA* were identified to be involved in significant biological pathways (*p*<0.05). (B) Predicted signaling pathways of all upregulated and downregulated differentially expressed genes (DEGs) without a protein-protein interaction network analysis. The top 10 genes of *PDGFA*, *LPAR1*, *IL6*, *COL4A6*, *COL4A5*, *LAMB3*, *RASGRP1*, *IL20*, *IL18R1*, and *INHBA* were identified to be involved in significant biological pathways (*p*<0.05).

We utilized 82 DEGs to predict protein-protein interactions using STRING software, and obtained 79 PPI pairs to construct PPI networks. All of the 79 PPI pairs were imported into Cytoscape software to map the PPI network and to determine hub proteins. As shown in Fig. 1C, we illustrated 79 significantly correlated protein network interactions (*p*<0.05) and marked totally 28 upregulated (red nodes) and downregulated DEGs (green nodes). A hub protein was defined as one having a degree cutoff of ≥1 and was simultaneously differentially expressed between parental cell lines and paclitaxel-resistant cell lines. Among 79 nodes, we determined 28 hub genes (Fig. 2, Supplementary Table 3, 4) and further predicted their signaling pathways and validated their clinical importance.

### KEGG pathway analysis

**Figure 3:**
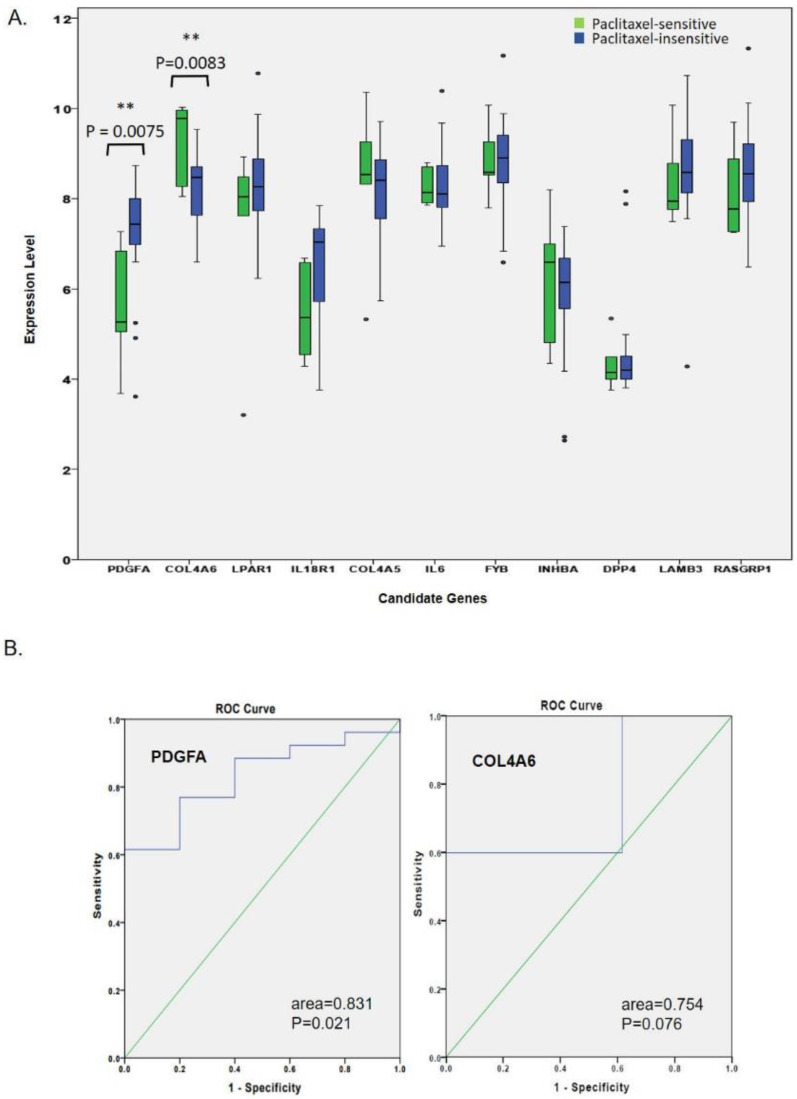
Expression levels of hub genes related to the paclitaxel response in triple-negative breast cancer (TNBC) patients. (A) In the GSE25065 mRNA analysis, clinical data of 31 patients treated with neoadjuvant paclitaxel-based reagents showed that five patients were sensitive to paclitaxel-based treatment, while 26 patients were insensitive. Only the upregulated *PDGFA* and downregulated *COL4A6* genes were significantly associated with an insensitive response to paclitaxel-based therapy. (B) An ROC curve analysis showed that the expression of *PDGFA*, but not of *COL4A6,* had excellent discrimination in predicting the response to paclitaxel.

We utilized the DAVID to classify and annotate the 28 hub DEGs from the PPI network analysis and also all DEGs without a PPI network analysis according to the KEGG pathway. Pathway predictions of 28 genes with a PPI analysis was shown in Fig. 2A and Supplementary Table 5. The top eight pathways reached statistical significance (*p*<0.05) included the cytokine-cytokine receptor interactions, the PI3K-Akt signaling pathway, pathways in cancer, the AGE-RAGE signaling pathway in diabetic complications, amoebasis, protein digestion and absorption, focal adhesion, and the Rap1 signaling pathway. Nine genes (*COL4A6*, *COL4A5*, *IL6*, *PDGFA*, *LPAR1*, *FYB*, *IL20*, *IL18R1,* and *INHBA*) were found involved in those pathways (Fig. 2A, Supplementary Table 5). In addition, we performed significant pathway predictions of all DEGs without a PPI analysis and then compared them with above results. There were 6 similar signaling pathways except small cell lung cancer and ECM-receptor interaction among the 8 pathways predicted. (Fig. 3B, Table 1). Furthermore, by comparing two KEGG pathways (Fig. 2A, B) using different analytical methods, two additional genes, *LAMB3* and *RASGRP1* were predicted from both Figs. 2A and 2B (Fig. 3B), while *FYB* was only predicted from Fig. 2A. Taken together, we identified 12 hub genes involved in paclitaxel resistance related signaling pathways: *COL4A6*, *COL4A5*, *IL6*, *PDGFA*, *LPAR1*, *FYB*, *IL20*, *IL18R1*, *INHBA*, *DPP4*, *LAMB3*, and *RASGRP1* (Fig. 3).

### Verification of candidate genes in TNBC patients' paclitaxel responses

We further attempted to validate whether or not the expression levels of the 11 hub genes were associated with a paclitaxel response. In the GSE25065 mRNA analysis, we compared expression levels of 11 hub genes in 31 TNBC patients (five sensitive and 26 insensitive responses) after they received neoadjuvant paclitaxel-based chemotherapy. As shown in Fig. 3A, only up-regulated *PDGFA* and down-regulated *COL4A6* were significantly associated with insensitive responses to paclitaxel-based therapy. Rest of the genes did not show a significant difference in TNBC patients' responses, and expression data for *IL20* in the GSE25065 dataset was not available. The expression trends for *LPAR1*, *IL18R1*, *COL4A5*, *FYB*, *LAMB3*, and *RASGRP1* were correlated with therapeutic responses. Among the paclitaxel-insensitive group, although with high variations in the genes expression levels,* LPAR1*, *IL18R1*, *FYB*, *LAMB3*, and *RASGRP1* seemed to have higher median expression levels, and* COL4A5,* a lower median expression level (Fig. 3A, Table 2). The ROC curve analysis also showed that the expression of *PDGFA* had an excellent discrimination (area=0.831, *p*=0.021) for predicting paclitaxel resistant response, while the expression of *COL4A6* did not (area=0.754, *p*=0.076) (Fig. 3B).

### Genes correlated with TNBC patient survival

**Figure 4:**
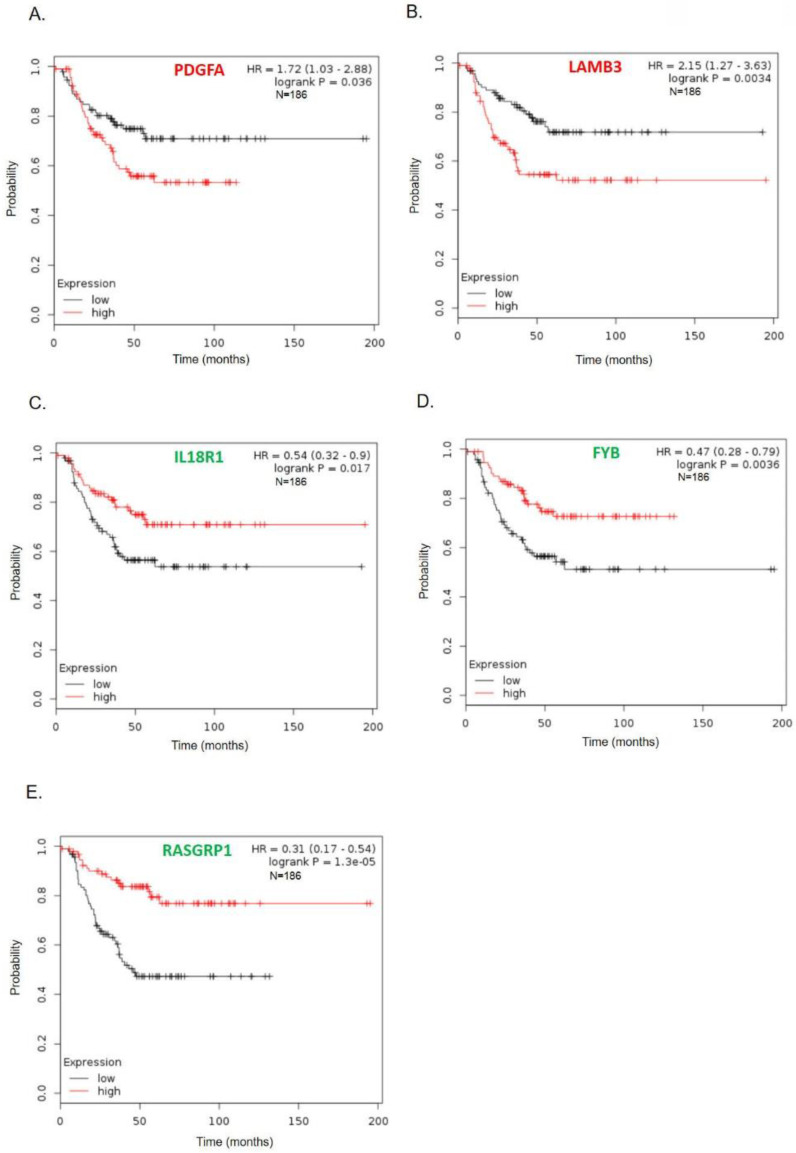
Kaplan-Meier recurrence-free survival curves present the prognosis being associated with expression levels of specific genes involved in paclitaxel resistance. (A) *PDGFA*, (B) *LAMB3*, (C) *IL18R1*, (D) *FYB*, (E) *RASGRP1IL6* (*n*=186). High expression levels of *PDGFA* and *LAMB3* were significantly correlated with poor recurrence-free survival, while patients with low levels of *FYB*, *IL18R1*, and *RASGRP1* tended to have significantly worse relapse-free survival.

To further survey the prognostic values of these 11 hub genes in TNBC, we analyzed associations of these genes with 186 TNBC patient's long-term regression-free survival. As shown in Fig. 4, high expressions of *PDGFA* and *LAMB3* were significantly correlated with poor recurrence-free survival (Figs. 4A-B), while patients with low levels of *FYB*, *IL18R1*, and *RASGRP1* tended to have significantly poorer relapse-free survival (Figs. 4C-E). Survival curves of other genes (*COL4A6*, *COL4A5*, *IL6*, *LPAR1*, *IL20*, *INHBA*, and *DPP*) did not show a significant difference either in the high or low gene expression groups (supplement Fig. 1).

## Discussion

TNBCs are vastly heterogeneous tumors and exhibit different responses to chemotherapy 26, and around 80 % of TNBC patients are basal-like subtype 11. A previous study showed that basal-like tumors with the higher proliferative characteristics exhibited greater sensitivity (around 40 percent pCR) to taxane-based chemotherapy, compared with luminal AR (LAR) tumors with the lowest Ki-67 index (around 20 percent pCR) 26. However, in our study, we focus on basal-like TNBC (intrinsic subtype) which included almost all BL1, BL2 and M. Non-basal like TNBC are composed from LAR. Our study provides some clues to explain the mechanism of taxane-based therapy resistance. We attempted to determine possible candidate genes involved in taxane-based therapy resistance especially for basal like TNBC.

Members of the platelet-derived growth factor (PDGF) family are major mitogenic signals for mesenchymal cells 28. PDGFs and vascular endothelial growth factors (VEGFs) are potential metastatic factors in breast cancer 29. The protein encoded by the *PDGFA* gene in humans is PDGF subunit-α 30. This gene product can exist as either a homodimer or heterodimer with PDGF-β 31. Overexpression of PDGF-α in breast cancer is associated with tumor progression in breast cancer 32. Inhibition of PDGF receptor (PDGFR) signaling restricted the bony metastasis of breast cancer in an animal model 33. Secretion of PDGF-α by a tumor resulted in recruitment of VEGF-producing fibroblasts, thereby reinforcing interactions between tumor cells and stromal cells through growth factors 34. PDGFs and PDGFRs are major players in tumorigenesis and drug resistance, and may be attractive oncologic targets in cancers 35, 36. PDGF mediates breast cancer cell desmoplasia 37 and the endothelial-mesenchymal transformation in glioblastomas and breast cancer 38, 39. In addition, PDGF increases cell proliferation and angiogenesis of luminal breast cancer via the PDGF/AKT signaling pathway 40, 41. In our research, we demonstrated high expression of *PDGFA* was significantly correlated with overall survival and poor recurrence-free survival. Taken together, *PDGFA* may be a poor prognostic marker of basal-like TNBC and is an excellent discriminator of paclitaxel resistance.

*COL4A5* and *COL4A6* encode two of the six subunits of type IV collagen 42, 43. The type IV collagen family consists of the major components of the basement membrane, which is important in confining the tumor microenvironment 44. Deletion of *COL4A6-COL4A5* is related to molecular pathogenesis of uterine leiomyomas 45, diffuse esophageal leiomyomatosis 46, and Alport's syndrome 47. Downregulation of *COL4A5* and *COL4A6* was correlated with metastasis in different cancers including melanomas, colorectal cancer, follicular thyroid cancer, prostate cancer, basal cell carcinoma, and breast cancer 48-53. TNBC-associated long non-coding (lnc) RNAs may target *COL4A6*, which can influence the onset and progression of TNBC 54. Our results showed that downregulation of *COL4A5* and *COL4A6* were related to paclitaxel resistance in TNBC. Downregulation of *COL4A6* was significantly associated with an insensitive response to paclitaxel-based therapy.

Upregulation of *LPAR1*, *FYB*, *INHBA*, *LAMB3*, and *RASGRP1* was found in paclitaxel-resistant cell lines after the KEGG pathways analysis, but no significance of those genes can be confirmed with paclitaxel-resistance on TNBC patients (Fig. 3A). Analysis of long-term regression-free survival showed that high expression of *LAMB3* and low levels of *FYB* and *RASGRP1* tended to be associated with a significantly worse relapse-free survival in basal-like TNBC patients. In addition, downregulation of *LPAR1* was the only hub DEG investigated related to poor survival in all TNBC patients but not in basal-like TNBC patients, implying that *LPAR1* might also play an important role in non-basal like TNBC (Supplementary fig.1). The *LPAR1* gene encodes a protein known as LPAR1 (Lysophosphatidic acid receptor 1) 55. G protein-coupled receptors are utilized by LPA (Lysophosphatidic acid) for cell signaling, mediating cell proliferation, platelet aggregation, muscle contraction, chemotaxis, and tumor cell invasion 56. A previous study described how LPAR1 plays a key role in tissue fibrosis and carcinogenesis 57 and modulates metastasis in basal breast cancer by activating the LPA1/ZEB1/miR-21 pathway 58.

*RASGRP1* is a guanine nucleotide exchange factor that activates RAS and the ERK/MAPK cascade 59, which is important for lymphocyte development and differentiation 60. A previous study showed that higher expression of *RASGRP1* was related to better overall survival and disease-free survival in breast cancer 61. In addition, a higher expression level of *RASGRP1* was correlated with better clinical outcomes for colorectal cancer (CRC) 62. A trend of higher *RASGRP1* expression was seen in paclitaxel-insensitive TNBC patients, which is consistent with our findings. Low *RASGRP1* expression was correlated with worse relapse-free survival in TNBC patients. Further study is needed to clarify the reverse role that *RASGRP1* plays in the response to paclitaxel and docetaxel treatment.

*FYN* encodes a membrane-associated tyrosine kinase which interacts with the FYN-binding protein and is related to control of cell growth 63. FYN can drive morphologic transformation and can increase “anchorage-independent growth and morphologic changes" 64, 65. Overexpression of *FYN* results in reduced sensitivity to tamoxifen treatment, and knockdown of *FYN* can restore sensitivity to tamoxifen in parental MCF-7/S0.5 cells 66, 67. FYB (FYN-binding protein) is an adapter for the FYN protein and is involved in platelet activation and control of interleukin (IL)-2 68. We found that overexpression of *FYB* was associated with paclitaxel resistance in basal-like TNBC cell lines, but *FYB* did not exhibit an impact in paclitaxel-treated patients.

*IL18R1* and *LAMB3,* two upregulated DEGs in paclitaxel-resistant cell lines, had trends of higher median expressions in paclitaxel-insensitive TNBC patients. High expression of *LAMB3* and low expression of *IL18R1* were significantly correlated with poor recurrence-free survival. The result of *LAMB3* is consistent with our analysis. However, that of *IL18R1* was controversial with the results obtained. In humans, the *LAMB3* gene encodes the beta 3 subunit of laminin 69, which refers to a family of basement membrane proteins. Extracellular laminin family members are also involved in tissue differentiation, wound healing, and tumorigenesis. Promoters of laminin family members, especially *LAMB5,* were described as being unmethylated in normal tissues and prone to abnormal methylation in breast cancer 70.

To sum up, our data showed that Upregulation of PDGFA and downregulation of COL4A6 were significantly associated with an insensitive response to paclitaxel-based therapy. In terms of prognosis, high expressions of *PDGFA* and *LAMB3* were significantly correlated with poor recurrence-free survival, while low levels of *FYB*, *IL18R1*, and *RASGRP1* tended to produce significantly worse relapse-free survival. A limitation of the recurrence-free survival analysis in this study was the high variations of patients' backgrounds, including heterogenous chemotherapy, since the combined data from different GEO databases was used. Further studies are required to resolve the the specific functions and mechanisms of these DEGs involved in taxane resistance. These dysregulated mRNAs might be promising biomarkers for predicting the response and prognosis of taxane therapy in patients with basal-like TNBC.

## Supplementary Material

Supplementary figures and tables.

## Figures and Tables

**Table 1:** Predict the signaling pathway of all DEGs without protein-protein interaction network analysis by DAVID.

KEGG Pathway	Genes	P-value	Adjusted p-value
Pathways in cancer (hsa05200)	PDGFA, LPAR1, IL6, COL4A6, COL4A5, LAMB3, RASGRP1	0.0013	0.066
Amoebasis (hsa05146)	IL6, COL4A6, COL4A5, LAMB3	0.00079	0.066
PI3K-Akt signaling pathway (hsa04151)	PDGFA, LPAR1, IL6, COL4A6, COL4A5, LAMB3	0.0029	0.09
Cytokine-cytokine receptor interaction (hsa04060)	IL6, PDGFA, IL20, IL18R1, INHBA	0.0049	0.09
Small cell lung cancer (hsa05222)	COL4A6, COL4A5, LAMB3	0.0055	0.09
AGE-RAGE signaling pathway in diabetic complication (hsa04933)	COL4A6, COL4A5, IL6	0.0085	0.091
ECM-receptor interaction (hsa04512)	COL4A6, COL4A5, LAMB3	0.0048	0.09
Protein digestion and absorbation (hsa04974)	COL4A6, COL4A5	0.0062	0.09
Focal adhesion (hsa04510)	COL4A6, COL4A5, PDGFA, LAMB3	0.0099	0.091

**Table 2:** Validate the genomic predictor of response to neoadjuvant chemotherapy from comparison of 5 paclitaxel- sensitive TNBC patients and 26 paclitaxel-insensitive TNBC patients in GSE25065 dataset.

Gene ID	Gene Name	log_2_ FC*^a^*	P value
**COL4A6**	collagen type IV alpha 6 chain	-0.984323	0.008318**
**COL4A5**	collagen type IV alpha 5 chain	-0.140993	0.793143
**IL6**	interleukin 6	-0.0487922	0.8896291
**PDGFA**	platelet derived growth factor subunit A	1.5646	0.007547**
**LPAR1**	lysophosphatidic acid receptor 1	1.12668	0.065488
**FYB**	FYN binding protein	0.66493	0.269866
**IL18R1**	interleukin 18 receptor 1	1.02107	0.075116
**INHBA**	inhibin beta A subunit	-0.372078	0.549308
**DPP4**	dipeptidyl peptidase 4	0.14094	0.774685
**RASGRP1**	RAS guanyl releasing protein 1	0.4179364	0.4161271
**LAMB3**	laminin subunit beta 3	0.1541256	0.77809559

***^a^*log_2_ FC**: expression fold change of insensitive over sensitive groups based on log_2_. *P value < 0.05, **P value < 0.01
