# Supplementary material for: Identified the novel resistant biomarkers for taxane-based therapy for triple-negative breast cancer
Source: Int J Med Sci. 2021 Apr 26;18(12):2521–31. doi: 10.7150/ijms.59177 (PMC8176163; doi:10.7150/ijms.59177)
Supplement: Supplementary file 1 — Supplementary figures and tables. [file ijmsv18p2521s1.pdf]

Supplementary table 1. Up-expression differentially expressed genes (DEGs) in paclitaxel resistant MDA-MB-468 and SUM149 from analyzing expression data of GSE90564.

Gene ID	MDA-MB-468 (logFC)	SUM-149 (logFC)
SERPINB2	6.59435124	3.79917612
ABCB1	7.65692508	10.62457819
PRSS12	4.96911212	1.5051214
SERPINB8	5.17488109	1.1527134
FAM171A1	3.56878183	1.33321409
MSX1	6.30427204	1.37241372
LOC728392	3.83423801	2.68497498
RYR2	4.01531378	3.21490947
STEAP1	3.00678615	2.2393608
CCDC3	4.17115951	1.46008096
KLK1	4.55614974	2.09289326
KCNG1	3.1532211	1.5237624
SERPINE2	3.54256713	1.20776762
DFNA5	2.95591583	1.28630132
CROT	2.60517102	1.94403633
VWA5A	3.23768455	1.13546624
CYP24A1	4.99812523	2.83884528
TEX15	2.35095698	1.00493828
C19orf51	2.7317467	1.45674104
KCNG1	3.1532211	1.5237624
SLCO4A1	3.3146991	1.17918092
KIAA1462	3.45062201	1.94005383
ALDH1A1	5.98099075	1.33122179
NCAM1	5.68822596	1.40480745
NKAIN1	2.62842987	1.25211691
IGF2BP1	2.81697537	1.30817216
INHBA	3.18307355	1.54179653
ABCB4	2.34024674	3.12721387
SPRR2A	3.92193559	1.82330734
TGM2	2.41239135	1.12206523
SRPX	2.88555796	1.52431657
HOXB9	2.49981422	2.84030054
RRAGD	3.01309192	1.82948467

NAT8L	3.3282139	2.0467167
SALL4	1.91349705	1.4872668
IL20	2.24388821	1.98644946
LAMB3	2.62368225	1.08433056
APOBEC3C	1.78426237	1.36125995
KCNJ12	2.08182696	1.86774994
FYB	1.85304731	1.75650501
SSTR2	3.79369433	1.38234365
APOBEC3F	1.7538556	1.31762012
CD22	3.14205459	1.34072024
HS3ST3B1	2.65632103	1.26542837
CLDN1	2.40275104	2.00377268
RGMA	1.91850179	1.06192543
RASGRP1	1.75607739	2.17714041
HERC6	2.29318812	1.17364571
CTHRC1	2.56580353	1.71631786
WIPF1	1.66392242	1.05532665
HOXB6	1.9301132	2.34268893
SOBP	2.2728673	1.36745799
SLC7A2	2.27540327	1.38107068
LPAR1	1.98245809	2.8882123
IL18R1	1.47771931	2.32407878
ZBED2	3.85060232	1.06830609
PDGFA	1.44389895	1.08243718
GBX2	1.95180886	1.57508779
PIWIL4	1.83217019	1.31211316
RAB3B	2.26320599	1.12321262
OLFML2A	1.79440695	1.47693924
ANKRD29	1.17807402	1.36307952
GFPT2	1.20644649	1.62286417
AGPAT9	1.54900402	2.38169087
DPP4	2.65838079	1.76056947
SEMA3A	1.97186181	4.11351805
IL6	1.97070095	2.98037819
PDGFRL	1.2861555	1.29692031
KBTBD11	1.97095834	1.54060436
APOBEC3B	1.30148122	1.33817593
HOXA11AS	2.31297568	1.22432624

Supplementary table 2. Down-expression differentially expressed genes (DEGs) in paclitaxel resistant MDA-MB-468 and SUM149 from analyzing expression data of GSE90564.

Gene ID	MDA-MB-468 (logFC)	SUM-149 (logFC)
CFI	-2.19929599	-3.99565753
CXXC4	-2.93068844	-2.36415485
COL4A5	-1.91104575	-1.62059115
OPHN1	-1.82406385	-1.45386335
ARSD	-2.68428442	-1.49096035
FRY	-2.72767195	3.93374879
COL4A6	-2.40328617	-1.08699208
FOXO4	-4.45304922	-1.06082997
ANKS1B	-2.26105986	-1.02427889
FBP1	-1.8160282	-1.48112138
RNASET2	-1.32233312	-1.00323545

Supplementary table 3. Protein-protein interaction (PPI) analysis to construct the network and predict the degree, betweenness and closeness of protein connection.

Gene ID	Degree	Betweenness	Closeness
PDGFA	1.0	0.0	0.05108055
GFPT2	1.0	0.0	0.050128534
NAT8L	1.0	0.0	0.050128534
NEO1	1.0	0.0	0.013497145
NCAM1	1.0	0.0	0.012984851
ADH1A	1.0	0.0	0.012984851
ADH4	1.0	0.0	0.012984851
COL4A6	1.0	0.0	0.012984851
SKAP2	1.0	0.0	0.048118446
PIWIL4	1.0	0.0	0.012820513
TDRD9	1.0	0.0	0.012820513
GPAM	1.0	0.0	0.012820513
AGPAT9	1.0	0.0	0.012820513
RGMA	2.0	8.0	0.0135064935
ALDH1A1	2.0	2.0	0.012987013
COL4A5	2.0	2.0	0.012987013
FYB	2.0	122.0	0.05
SERPINE2	2.0	0.0	0.053133514
HERC6	2.0	0.0	0.052489907
SERPINB2	2.0	0.0	0.052173913
SERPINB8	2.0	0.0	0.052173913
GIP	2.0	0.0	0.052525252
CFI	2.0	0.0	0.050518136
CFH	2.0	0.0	0.050518136
WASL	3.0	66.62857	0.052953158
DPP4	3.0	464.0	0.05266712
CAD	3.0	242.0	0.052173913
LCP2	3.0	240.0	0.051965356
WIPF1	3.0	19.0	0.052139036
PDGFRA	3.0	122.0	0.053206004
IL20RB	3.0	11.292135	0.053315107
IL20RA	3.0	11.292135	0.053315107
FOXO4	3.0	1.1666666	0.054054055
IL18RAP	3.0	0.0	0.05338809

SALL4	3.0	0.0	0.053133514
NANOG	3.0	0.0	0.053133514
SSTR2	3.0	0.0	0.052489907
TRDN	3.0	0.0	0.051349573
FKBP1B	3.0	0.0	0.051349573
ACVR2A	3.0	0.0	0.0135064935
INHBA	3.0	0.0	0.0135064935
ACVR2B	3.0	0.0	0.0135064935
NRP1	3.0	0.0	0.04890282
SEMA3A	3.0	0.0	0.04890282
PLXNA1	3.0	0.0	0.04890282
IL18R1	4.0	6.2788463	0.053608246
RYR2	4.0	48.666668	0.05227882
WAS	4.0	461.1476	0.05397924
IL20	4.0	4.0	0.05193076
PLXNA4	4.0	354.0	0.05078125
POU5F1	4.0	32.91111	0.05397924
ASPH	4.0	210.27953	0.05338809
CALM1	4.0	138.88715	0.054204307
FST	4.0	12.0	0.0135111725
RRAGB	4.0	0.0	0.05357143
RRAGD	4.0	0.0	0.05357143
RRAGA	4.0	0.0	0.05357143
SST	5.0	99.18315	0.05394191
C3	5.0	240.0	0.052560646
RPTOR	5.0	12.933333	0.053793103
UBA52	6.0	491.1461	0.054242004
IL6ST	6.0	36.070213	0.053682037
IL17A	6.0	1.6799145	0.05397924
GCG	7.0	713.4315	0.054583624
IL6R	7.0	61.035667	0.05394191
IL18	7.0	25.443657	0.05401662
LPAR1	8.0	407.51294	0.054583624
PLAU	8.0	272.7635	0.0543554
F2	8.0	224.07857	0.055123676
SOCS3	8.0	101.34484	0.05394191
IL1B	9.0	43.321095	0.054890923
JUN	12.0	461.75378	0.0554371

STAT1	12.0	181.58154	0.055123676
MAPK1	12.0	175.49641	0.055045873
FOS	13.0	458.59244	0.055634808
NFKB1	15.0	558.9963	0.055634808
IL6	17.0	554.8925	0.055516016
STAT3	17.0	504.69122	0.05535841
AKT1	17.0	1184.501	0.05575411

Supplementary Table 4. The connected proteins (28 hub nodes) identified from differentially expressed genes (DEGs) analysis and Protein–protein interaction (PPI) network construction.

Gene ID	MDA-MB-468 (logFC)	SUM-149 (logFC)
**up-regulated DEGs**		
RGMA	1.91850179	1.06192543
IL18R1	1.47771931	2.32407878
IL6	1.97070095	2.98037819
RYR2	4.01531378	3.21490947
DPP4	2.65838079	1.76056947
LPAR1	1.98245809	2.8882123
IL20	2.24388821	1.98644946
ALDH1A1	5.98099075	1.33122179
FYB	1.85304731	1.75650501
RRAGD	3.01309192	1.82948467
SALL4	1.91349705	1.4872668
SERPINE2	3.54256713	1.20776762
HERC6	2.29318812	1.17364571
SSTR2	3.79369433	1.38234365
SERPINB2	6.00647273	3.79917612
SERPINB8	5.17488109	1.1527134
INHBA	3.18307355	1.54179653
SEMA3A	1.97186181	4.11351805
PDGFA	1.44389895	1.08243718
GFPT2	1.20644649	1.62286417
NAT8L	3.3282139	2.0467167
NCAM1	5.68822596	1.40480745
PIWIL4	1.83217019	1.31211316
AGPAT9	1.54900402	2.38169087
**down-regulated DEGs**		
COL4A5	-1.91104575	-1.62059115
FOXO4	-4.45304922	-1.06082997
CFI	-2.19929599	-3.99565753
COL4A6	-2.40328617	-1.08699208

Supplementary table 5. Predict the signaling pathway of 28 candidate genes with protein-protein interaction network analysis by DAVID.

KEGG Pathway	Genes	P-value	Adjusted p-value
**Cytokine-cytokine receptor interaction (hsa04060)**	IL6, PDGFA, IL20, IL18R1, INHBA	3.011E-05	2.259E-03
**PI3K-Akt signaling pathway (hsa04151)**	COL4A6, COL4A5, IL6, PDGFA, LPAR1	9.959E-05	3.3735E-03
**Pathways in cancer (hsa05200)**	COL4A6, COL4A5, IL6, PDGFA, LPAR1	2.027E-04	4.67E-03
**AGE-RAGE signaling pathway in diabetic complication (hsa04933)**	COL4A6, COL4A5, IL6	3.376E-04	4.67E-03
**Amoebasis (hsa05146)**	COL4A6, COL4A5, IL6	3.363E-04	4.67E-03
**Protein digestion and absorbation (hsa04974)**	COL4A6, COL4A5	2.661E-03	4.67E-03
**Focal adhesion (hsa04510)**	COL4A6, COL4A5, PDGFA	0.002761	0.02301
**Rap1 signaling pathway (hsa04015)**	PDGFA, LPAR1, FYB	0.003122	0.02342

supplement Fig1

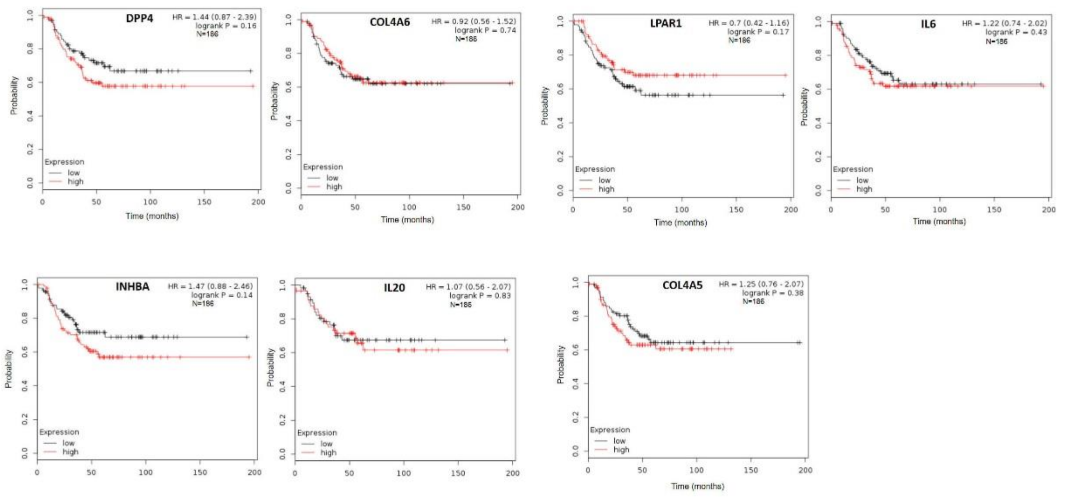

Supplemental Fig1. Kaplan-Meier recurrence-free survival curves presenting the prognosis associated with expression levels of specific genes involved in paclitaxel resistance. DPP4, COL4A6, LPAR1, IL6, INHBA, IL20, and COL4A5 (n=186) didn’t show significance in patient’s survival.
